# Correcting the pandemic: Analysis of corrections to journal articles on COVID-19 and Ebola

**DOI:** 10.7189/jogh.10.020105

**Published:** 2020-12

**Authors:** Jakov Matas, Ivan Buljan, Ana Marušić

**Affiliations:** Department of Research in Biomedicine and Health, University of Split School of Medicine, Split, Croatia

COVID-19, declared by the World Health Organization (WHO) as public health emergency of international concern on 30 January 2020 and as pandemic on 12 March 2020 [1], has brought unprecedented interest in research about this disease, resulting in massive publication output and shortened time to publish [2]. The need for rapid communication of research puts pressure on journals to balance rigorous review process with publication speed. Recent retractions in two major medical journals [3] have put doubt on the ability of journals to deal with these pressures. Some journals have already warned about the quality of evidence on COVID-19 [4] and there is concern that poor quality research on COVID-19 presents research waste and prevents pandemic response based on evidence [5].

We analyzed corrections to COVID-19 literature in the first five months of 2020, and compared them to those on Ebola, a recent public health emergency of international concern [6]. We searched PubMed using terms “COVID-19” (1 January-31 May 2020) and “Ebola” (1 August-31 December 2014) to retrieve relevant publications and their corrections in five months around the declaration of public emergency. We also searched CrossRef database for corrections to the retrieved articles, and Retraction Watch database (https://retractionwatch.com/retracted-coronavirus-covid-19-papers/). Two authors checked the correction/retractions notices and relevant articles in PubMed and at journals’ websites. The search strategy and the list of identified corrections and retractions are available in OSF (https://osf.io/nkp4t).

In the five months surrounding the declaration of a global public health emergency, there were 18 863 published items for COVID-19 and 962 for Ebola (**Table 1**). We identified 78 corrected or retracted articles on COVID-19 (0.4%) and 7 corrected articles on Ebola (0.7%). Most of them were research articles, and the corresponding authors mostly came from Asia for COVID-19 articles. Corrections to the articles on COVID-19 were published in 57 different journals and in 6 journals on Ebola. The impact factor of journals publishing corrections to COVID-19 articles was higher than those publishing articles on Ebola (median = 9.193, 95% confidence interval (CI) = 5.873-19.315 vs 3.570, 95% CI = 1.817 to 27.604).

**Table 1:** Corrections of articles published about COVID-10 (n = 18 863) and Ebola (n = 962)*

Characteristics	COVID-19	Ebola
**Number of corrected articles** (% of total published articles)	78 (0.4)	7 (0.7)
**Number of corrections/retractions†**	73/7	7/0
**Type of corrected/retracted articles:**		
Research article/systematic review	33	2
Review article	11	0
Other (letter to the editor, commentaries, opinion pieces, reports, etc.)	34	5
**Geographical origin of corrected/retracted articles (corresponding author address): ‡**		
Europe	24	2
Asia and Australia	33	1
Africa	1	1
South America	1	0
North America	19	3
**Reasons for correction:**		
Data (including figures and tables)§	53	6
Bibliographical information	12	1
Authorship	4	0
Combination of above	4	0
**Reasons for retraction:**		
Data (including figures and tables)¶	5	0
Not stated	2	0

*Time-frame for the articles: COVID-19: 1 January 2020 to 31 May 2020, Ebola: 1 August 2014 to 31 December 2014.

†One COVID-19 article was first corrected and then retracted; and one was corrected twice.

‡21 articles on COVID-19 originated from China; USA was the country of origin for 18 COVID-19 articles and all three Ebola articles.

§Reasons for data correction: change in numerical data (n = 13 for COVID-19), change in non-numerical data (n = 35 for COVID-19, 6 for Ebola), both numerical and non-numerical data (n = 4 for COVID-19), not clear (n = 1 for COVID-19).

¶Reasons for data retractions: authors falsely claiming first-hand experience (n = 1), incorrect data calculation (n = 1), inability to validate primary data sources (n = 2), results and conclusions bases on theoretical deduction and not field epidemiology data (n = 1).

The median time between article publication and its correction was 22 days (95% CI = 15-27) for COVID-19 and 82 days (95% CI = 1-87) for Ebola. Reasons for corrections were mostly changes to data (56 for COVID-19 and 10 on Ebola), predominantly non-numerical (36 for COVID-19, none for Ebola) (**Table 1**). 24 of the corrected or retracted COVID-19 articles included patients (median number of patients = 32, 95% CI = 4-275). There were no studies involving patients among corrected articles on Ebola. Most of the corrections notices were signed by the authors (n = 35 for articles on COVID-19 and n = 3 for articles on Ebola) or there was no statement (n = 42 and n = 4, respectively); in 3 cases for COVID-19 articles a journal signed the statement. Corrected articles for both topics were mostly adequately indexed in PubMed: 71 were “published erratum” for articles on COVID-19 and 7 for those on Ebola. For articles on COVID-19 there were 5 “retractions of publication”, 1 “addendum” and for 3 articles the corrections were not indexed as such (one correction from a journals was not indexed, and in two cases the original articles were not indexed but the indexed version had “retracted” or “withdrawn” in the title.

The number or corrected articles on COVID-19 indicate the haste in publishing, which is not surprising considering the volume and the unprecedented interest of the research community and the public. In comparison to Ebola, as the other most recent public emergency of international concern, publication surge about COVID-19 resulted in comparable literature corrections.

Shorter time to correct or retract information in COVID-19 articles than those in Ebola articles may mean that both authors and editors behaved responsibly in ensuring the integrity of the published record and provide reliable evidence base to make valid decision in addressing the disease and the pandemic. On the other hand, the finding that 9% of COVID-19 literature corrections were retractions, in comparison to none for articles on Ebola, indicates the pressure to publish may lead to research misbehavior.

This is just the first snapshot of the corrections to the research on COVID-19 pandemic. The pandemic continues (with more than 42 thousand published articles indexed in PubMed on August 20, 2020) and more corrections to the published literature are expected. We will be following them and assess how effective the scientific community was in ensuring reliable and valid evidence base for addressing the biggest global health emergency of this century.

As the COVID-19 pandemic continues, the research community should continue to adhere to the highest principles of research integrity in their work, the journals should continue to deal quickly and efficiently with correcting the published record, and bibliographical databases should consistently and rapidly record the corrections so that only valid evidence base can be used in health care.
